# Implicit Emotion Regulation Deficits in Trait Anxiety: An ERP Study

**DOI:** 10.3389/fnhum.2018.00382

**Published:** 2018-09-28

**Authors:** Bingqian Liu, Yi Wang, Xuebing Li

**Affiliations:** ^1^CAS Key Laboratory of Mental Health, Institute of Psychology, Chinese Academy of Sciences, Beijing, China; ^2^University of Chinese Academy of Sciences, Beijing, China

**Keywords:** implicit emotion regulation, anxiety, trait anxiety, N170, EPN, LPP

## Abstract

According to the framework of emotion regulation (ER), both explicit and implicit forms are essential to our well-being. It is the interaction between these two processes that ensures adaptive emotional responses. Although many studies have focused on explicit ER deficits in anxiety, there is still a lack of awareness about the implicit form and its role in anxiety. To address this issue, we explored the time course of implicit ER processes in individuals with high and low trait anxiety (LTA). To do this, we employed the newly developed Priming-Identify (PI) paradigm, which includes a word-matching task (externally-generated implicit goals) and a facial expression identification task (emotion processing). We aimed to modulate the implicit ER goals of individuals through the application of different priming conditions (ER-related and -unrelated words). In addition to their behavioral effects, we recorded the influence of these priming conditions through event-related potentials (ERPs) during the facial expression identification task. Three ERP components were chosen as indexes of three stages of implicit ER processing: N170, early posterior negativity (EPN) and late positive potential (LPP). In individuals with LTA, the early N170 and the middle EPN were enlarged under the ER-related priming condition, while the LPP was not influenced. However, in individuals with high trait anxiety (HTA), we observed an absence of any significant differences between the ER-related and -unrelated priming conditions across all three ERP components. Furthermore, enlargements of N170 and EPN amplitudes were significantly correlated with a decrease in negative emotion experience scores. Our results suggest that HTA individuals experience implicit ER deficits during the early and middle stages of ER.

## Introduction

With a global prevalence of 7.3%, anxiety disorders constitute the most prevalent class of mental disorder (American Psychiatric Association, 2013; Baxter et al., 2013). Although long considered disorders of emotion (Barlow, 1991; Beck and Emery, 2010), Kring (2010) noted that their most problematic and disruptive characteristic is not necessarily the content of the experienced negative emotion but rather its timing and intensity. This indicates the essential role played by emotion regulation (ER) in how individuals deal with anxiety. ER is generally defined as the application of abilities, methods or strategies “to influence which emotions we have, when we have them, and how these emotions are experienced or expressed” (Gross, 1998; Rottenberg and Gross, 2006). A growing body of research suggests that emotion dysregulation, the inability to regulate emotion, is heavily implicated in the etiology and maintenance of anxiety disorders (Mennin, 2004; Kashdan et al., 2008; Cisler et al., 2010; Suveg et al., 2010; Hofmann et al., 2012). Given these considerations, further investigation into ER deficits and specifically their role in anxiety disorders would contribute to an improved understanding of this disruptive class of disorders.

According to the dual-process framework of ER (Gyurak et al., 2011), adaptive emotional responses depend on an interaction between both explicit and the implicit forms of processing. However, the majority of studies investigating ER have focused solely on deficits of explicit ER in anxiety, that is, processes that require conscious efforts. These have demonstrated a tendency for anxious individuals to overuse maladaptive strategies, such as suppression, in place of more adaptive ER strategies, such as reappraisal (Berking et al., 2008; McLaughlin et al., 2011; Ball et al., 2013; O’Toole et al., 2014; Schäfer et al., 2017). Implicit ER processes, on the other hand, are evoked automatically by stimuli and occur without awareness. It is unreasonable to assume that explicit ER could be employed at all times, given the conscious effort required. As a consequence, the efficient use of implicit ER strategies is regarded as crucial to well-being (Gyurak et al., 2011). Despite this, there are few studies examining the role of implicit ER in anxiety.

Two behavioral studies suggest the presence of deficits in implicit ER in anxious individuals. In one study performed by Jasper and Witthöft (2013), participants were asked to perform a character valence attribution task. As a result, a significant correlation was found between the affect misattribution procedure and trait anxiety scores in a threatening priming condition. This finding suggests the presence of an automatic misattribution of negative affect related to anxiety, which in turn might indicate a deficit of implicit ER within these individuals. Ma and Zhu (2012) employed a sentence unscrambling task to investigate implicit ER in trait anxiety. Reappraisal-related words as well as suppression-related words were included in this task to exogenously modulate the implicit ER goal. In the subsequent affective picture labeling task, they found that, compared with healthy controls, high trait anxiety (HTA) individuals showed a significantly higher level of arousal in response to pictures with emotional content for both negative emotion and neutral emotion pictures under suppression-related priming and non-priming conditions, but a non-significant difference under the reappraisal-related priming condition. In their study, they found that emotional experiences could be modulated by priming ER goals effectively and high trait anxious individuals probably had the deficit of implicit suppression strategy.

While these studies indicate that anxious individuals might present deficits of implicit ER at the behavioral level, other studies have explored their underlying neural mechanisms. In two studies employing event-related potential (ERP) techniques, by applying the Go/Nogo paradigm to children with anxiety, have observed an enlarged Nogo trial-related N2 (Lamm et al., 2011) as well as significantly larger posterior P1 and frontal N2 amplitudes (Hum et al., 2013). Since both P1 and N2 occur in the early stage, these results suggest a deficit in early implicit ER processing. One functional magnetic resonance imaging (fMRI) study showed that healthy adults were able to regulate emotional conflict without awareness, however, an absence of this ability was observed in patients who were suffering from generalized anxiety disorder (Etkin et al., 2010).

The above studies suggest the presence of implicit ER deficits in anxious individuals at both behavioral and neural levels. However, one may argue that some of the deficits described above are more related to the process of emotion generation rather than ER. Some researchers argue that these two types of processing are inextricably entwined, and the regulatory process is a durable aspect accompanying the development of emotion over time (Amstadter, 2008). Given its complexity, a methodology that reveals the time course of ER during processing might provide more insight into the underlying mechanism of implicit ER as well as associated anxiety-related deficits. ERP is a temporally sensitive technique that could potentially offer such an insight. Although existing studies of anxiety have revealed various relevant ERP components that occur during emotion processing (e.g., P1, N2), it is difficult to determine whether those elicited by the Go/Nogo paradigm or during passive viewing are truly related to ER rather than the process of emotion generation.

In the current study, we adopted the operational definition given by Mauss et al. (2007a) to investigate the deficits occurring during the process of nonconscious goal pursuit in anxiety. According to this definition automatic/implicit ER is a “goal-driven change to any aspect of one’s emotions without making a conscious decision to do so, without paying attention to the process of regulating one’s emotions, and without engaging in deliberate control.” Thus, the mechanism underlying implicit ER is posited to be a nonconscious goal pursuit. Priming tasks, by their nature, offer the possibility of exogenously activating and manipulating the ER goals (e.g., emotion control and expression) of individuals without their conscious awareness. As a consequence, this can lead to the alteration of subsequent emotional experiences (Gallo et al., 2009; Williams et al., 2009; Yuan et al., 2015a).

Rather than evoking passive responses to specific emotional states, the recently developed Priming-Identify (PI) ERP paradigm (Wang and Li, 2017) was able to distinguish the emotional responses and ER processes. We adopted this method (Wang and Li, 2017) to explicitly alter implicit ER goals. We aimed to modulate them through two word-priming conditions in the word-matching component of the paradigm, namely ER-related and ER-unrelated priming. During this paradigm, after priming the ER goals, a facial expression identification task was used to investigate the alteration of emotional response processing, in which the electroencephalogram (EEG) signal was recorded. In order to ensure that this alteration was uniquely influenced by ER but not emotion generation, only threatening facial expressions (anger or fear) were presented during this task. In addition, an oral-based intention detection test was performed at the end of each testing session to ensure that the ER processing involved in this study was indeed implicit.

According to previous studies examining the time course of emotional facial processing (Frenkel and Bar-Haim, 2011; Fruhholz et al., 2011; Leleu et al., 2015; Wieser and Moscovitch, 2015), three ERP components are important indexes during this process: N170, early posterior negativity (EPN) and late positive potential (LPP). N170 is a negative-going component that occurs over occipital-temporal regions (Hinojosa et al., 2015). It is considered to be a face-specific ERP component which reflects the early, automatic perceptual process of face encoding (Bentin et al., 1996; Eimer and Holmes, 2002; Walentowska and Wronka, 2012). EPN is an occipital-temporal negative-going wave, which has been found to be enlarged for negative compared to neutral facial expressions during both implicit and explicit tasks (Morel et al., 2014; Yoon et al., 2016). LPP is a centro-parietal positive component, which represents more strategic high-level processes such as enhanced encoding of emotional expressions (Schupp et al., 2004b; Yuan et al., 2015b). The early window of LPP is considered to be an index of the allocation of attentional resources (Dennis, 2010).

It has been suggested that responses to emotional faces could be detected by the early N170 and selected for further encoding by the middle EPN, which may lead to elaborate processing at the level of LPP (Fruhholz et al., 2011). Accordingly, it is possible that implicit ER processing of facial expression could also be indexed by these components. One study has investigated the alteration of these ERP components under conditions designed to prime implicit ER. By exploring the time course of implicit ER of healthy adults through the PI paradigm, Wang and Li (2017) observed a more negative N170 (i.e., enlargement of N170 amplitude) induced by implicit ER, while the middle EPN and the late LPP were not influenced. Based on these findings, they suggest that the enlarged N170 could be considered an effective index of implicit ER. In the current context, we suggest that deficits of implicit ER in anxiety occur during the early stage of emotional facial processing and be detectable through the absence of an enlarged N170 amplitude.

Based on the above rationale, the aim of the current study was to investigate potential implicit ER deficits in individuals with HTA, as compared with individuals with low trait anxiety (LTA). Two hypotheses were raised in view of the previous literature. First, we hypothesized that in individuals with LTA, the early N170 would be enlarged by ER-related priming, while the middle EPN and the late LPP would remain unaffected. Second, in individuals with HTA, these three ERP components would not show any significant difference between the ER-related and -unrelated priming conditions. That is, an ER-related deficit would be present in the early stage of emotional facial processing for those individuals with HTA.

## Materials and Methods

### Participants

Thirty-six participants were selected from 570 college students in Beijing, China. The Spielberger Trait Anxiety Inventory (Spielberger et al., 1970) was applied during the pre-screening test. Participants with scores in the top 20% were allocated to the HTA group, and those with scores in the bottom 20% were allocated to the LTA group. Demographic information is presented in Table 1. All participants were right-handed, had normal or corrected-to-normal eyesight, and had no history of psychiatric or neurological disorders. All subjects gave written informed consent in accordance with the Declaration of Helsinki and were monetarily rewarded for their involvement. The research was approved by the local ethics committee (Institute of Psychology, Chinese Academy of Sciences).

**Table 1:** Demographic information for high trait anxiety (HTA) and low trait anxiety (LTA) groups.

Demographic information	HTA (*N* = 18)	LTA (*N* = 18)	*t*-value	*p*-value
Age (*M* ± SD)	20.33 ± 2.09	20.55 ± 2.62	−0.282	0.780
Gender (*n* male)	13	9	1.365	0.181
Years of education	14.06 ± 1.76	14.11 ± 2.14	−0.085	0.933
(*M* ± SD)				
STAI-T (*M* ± SD)	50.06 ± 3.61	29.11 ± 3.38	17.991	<0.001

### Stimulus Materials

In the current study, we used the same paradigm and stimulus materials as the study of Wang and Li (2017). First, for the word-matching task, two categories of priming words were selected for this study, namely ER-related (e.g., adjust, inhibit) and ER-unrelated (e.g., cancel, run) words. Priming words were evaluated and categorized by an independent group of Chinese students according to their meaning. The “ER-related” category corresponds to the “control” category in the previous study, whereby these words have been shown to effectively decrease negative emotion experience, that is, appropriate to induce implicit ER. “ER-unrelated” category corresponds to the previous “unrelated” category, all words within which has been shown to be not correlated with ER. The additional “expression” category in the prior study was not adopted in the present study because it has not been shown to effectively induce implicit ER. Second, for the facial expression identification task, all eighty face pictures were selected from the Chinese Facial Affective Picture System (CFAPS; Luo et al., 2010), including 40 fear faces and 40 anger faces. Male and female faces were equally presented in each condition. Differences in arousal between these two types of threatening facial expressions based on the CFAPS database was not significant (*t*_(78)_ = −0.940, *p* = 0.351). For more details, see Supplementary Information of the previous PI paradigm-based study (Wang and Li, 2017).

### Experiment Design and Procedure

The formal PI paradigm contained a word-matching task and a facial expression identification task. The word-matching task had two conditions according to the priming word categories (i.e., “ER-related” and “unrelated”). The facial expression identification task had two facial expressions, i.e., anger and fear. Participants were told that the aim of the study was to investigate the vocabulary processing and the emotional processing.

In the word-matching task, participants were asked to choose one word from a pair of words at the bottom of the screen that matched the meaning of the word presented at the top of the screen (i.e., find a synonym) by pressing the appropriate key. Participants were informed at the time of testing that the aim of this task was to check their vocabulary ability. In the facial expression identification task, a fixation cross was presented at the center of the screen for 200–500 ms prior to a picture of a face. Participants were required to judge whether the expression on the face was one of anger or fear as quickly as possible during its presentation (1,000 ms). The corresponding relation between emotion and appropriate key was balanced across subjects. Once the facial expression had left the screen, a 1,000 ms blank page was presented before the beginning of the next trial. During and after the facial expression identification task, participants were required to rate their level of negative emotion experience using a 9-point scale, every 30 trials.

**Figure 1:**
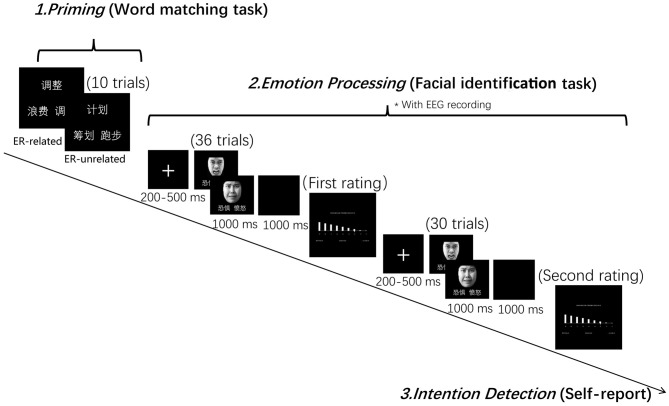
Experiment design and procedure. The Chinese characters mean “adjust,” “regulate” and “waste” in the emotion regulation (ER)-related condition, and “plan,” “arrange” and “run” in the ER-unrelated condition, as illustrated in the figure. Those two words repeatedly appeared in the facial identification task mean “Fear” and “Anger,” respectively.

The order of the two priming conditions was completely randomized across participants with each condition divided into three successive blocks. In each block, participants were required to complete 10 word matching trials, followed by 66 facial identification trials and two ratings of negative emotional experience (see Figure 1). In order to minimize the task switching effect, the first six trials of the expression identification task in each block were excluded from data analysis. An intention detection test was performed at the end of each testing session, during which participants were asked to report whether they had noticed a relationship between the word matching task and the facial identification task.

### EEG Recording

The EEG was recorded from 64 scalp sites using tin electrodes placed on an elastic cap (NeuroScan Inc., Herndon, VA, USA) with a left mastoid reference electrode (online) and were re-referenced (offline) to the mean of bilateral mastoid electrodes. All electrode impedance was maintained below 5 KΩ. EEG signals were amplified using a 0.05–100 Hz band-pass and sampled at the rate of 500 Hz. During the offline analysis, the EEG data were filtered with a low-pass at 30 Hz (12 dB/oct). The ERP data were segmented from −200 ms to 1,000 ms relative to the face onset in the expression identification task, with a baseline correction of 200 ms pre-stimulus. Any trials in which the EEG voltages exceeded ±80 μV were excluded from analysis.

### Data Analysis

Negative emotion experience scores were analyzed for two ordinal positions (first and second) during facial identification task prior to both priming conditions (ER-related and ER-unrelated). Reaction times from the facial expression identification task were calculated for correct trials prior to two priming conditions (ER-related and ER-unrelated). Trials were excluded if the reaction time was <200 ms or >1500 ms.

For ERP data, average amplitudes were overlaid for correct trials prior to the two priming conditions. We combined these two types of threatening faces together for the ERP analysis as there were no significant priming effect differences between fear and anger face processing. In line with the previous study (Wang and Li, 2017), eight electrode sites (P7, P5, P6, P8, PO7, PO5, PO6, PO8) were selected for N170 (145–190 ms) and EPN (250–320 ms), and nine (C3, CZ, C4, CP3, CPZ, CP4, P3, PZ, P4) for the analysis of LPP (450–750 ms).

All statistical analyses were performed using SPSS (17.0; SPSS, Inc., Chicago, IL, USA) with a significance level set at 0.05. The Greenhouse-Geisser correction was used to compensate for sphericity violations. Partial eta-squared ($\eta_{\text{p}}^{2}$) were reported as an indicator of the effect size in ANOVA tests, where 0.06 represents a medium effect and 0.14 a large effect (Cohen, 1988).

## Results

### Behavioral Results

#### Negative Emotion Experience

**Figure 2:**
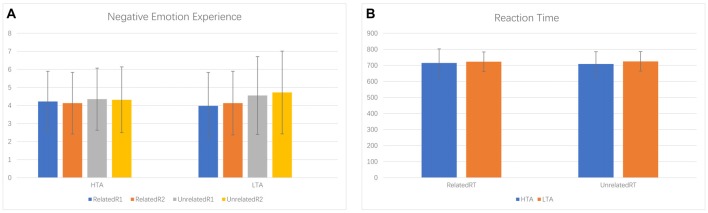
Bar plots for negative emotion experience **(A)** and reaction time **(B)**.

Negative emotion experience rating scores were analyzed by a 2 (priming conditions: ER-related/ER-unrelated) × 2 (ordinal positions: first rating/second rating) × 2 (group: HTA/LTA) repeated measures ANOVA. We observed a significant main effect of priming condition (*F*_(1,34)_ = 4.112, *p* = 0.050, $\eta_{\text{p}}^{2}$ = 0.108). Specifically, the ER-related priming condition (*M* = 4.115, SD = 0.286) was associated with a lower negative emotion experience score during the subsequent facial identification task than scores associated with the ER-unrelated priming (*M* = 4.486, SD = 0.331). Results also revealed non-significant main effects for ordinal positions (*F*_(1,34)_ = 0.243, *p* = 0.625) and group (*F*_(1,34)_ = 0.026, *p* = 0.872). Although we found no significant interaction between priming conditions and group (*F*_(1,34)_ = 1.315, *p* = 0.259), descriptive statistics did reveal a larger difference of negative emotion experience scores between ER-related and ER-unrelated priming within the LTA group (*M* = −0.581, SD = 1.263) than the HTA group (*M* = −0.161, SD = 0.902; see Figure 2).

#### Reaction Time

For the reaction time of facial identification task, a 2 (priming conditions: ER-related/ER-unrelated) × 2 (group: HTA/LTA) ANOVA indicated non-significant main effects of priming condition (*F*_(1,34)_ = 0.129, *p* = 0.721) and group effect (*F*_(1,34)_ = 0.264, *p* = 0.611). The interaction between priming and group was not significant (*F*_(1,34)_ = 0.406, *p* = 0.529; see Figure 2).

#### Intention Detection

No participants realized that the word matching task was aimed at regulating negative emotion in the subsequent task, based on the oral self-report of all 36 participants.

### ERP Results

#### N170 Amplitude

A 2 (priming conditions) × 2 (horizontal electrodes: P/PO) × 4 (vertical electrodes: 5/6/7/8) × 2 (group) repeated measures ANOVA was conducted for N170 amplitudes. We found a marginally significant main effect of priming condition (*F*_(1,34)_ = 3.175, *p* = 0.084, $\eta_{\text{p}}^{2}$ = 0.085), whereby ER-related priming elicited larger N170 (*M* = 0.246, SD = 0.426) than the ER-unrelated priming (*M* = 0.585, SD = 0.458). A significant main effect of vertical electrodes (*F*_(3,102)_ = 20.207, *p* < 0.001, $\eta_{\text{p}}^{2}$ = 0.373) was found, and the N170 at the right-side electrodes were significantly larger than left-side ones. We also observed a marginally significant interaction between priming condition and group (*F*_(1,34)_ = 3.981, *p* = 0.054, $\eta_{\text{p}}^{2}$ = 0.105). The main effects of horizontal electrodes (*F*_(1,34)_ = 0.643, *p* = 0.428) and group (*F*_(1,34)_ = 2.163, *p* = 0.151), however, were not significant.

**Figure 3:**
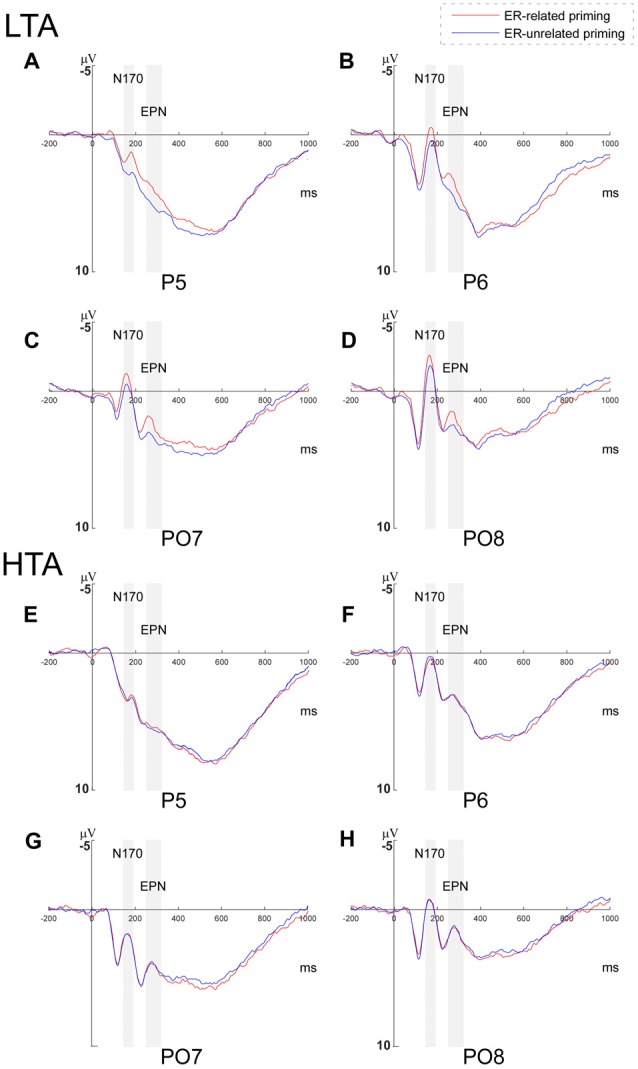
Grand averages of N170 and early posterior negativity (EPN) amplitudes. Grand averages of N170 and EPN amplitudes evoked during the Priming-Identify (PI) task at P5, P6, PO7 and PO8 within **(A–D)** the low trait anxiety (LTA) and **(E–H)** the high trait anxiety (HTA) groups.

Since the interaction between priming conditions and group was marginally significant, a further 2 (priming conditions) × 2 (horizontal electrodes: P/PO) × 4 (vertical electrodes: 5/6/7/8) repeated measures ANOVA was conducted separately within the HTA and the LTA group. In the HTA group, the main effect of priming was not significant (*F*_(1,17)_ = 0.118, *p* = 0.851), while in the LTA group, the analysis revealed a significant main effect of priming condition (*F*_(1,17)_ = 5.177, *p* = 0.036, $\eta_{\text{p}}^{2}$ = 0.233; see Figure 3).

##### EPN Amplitude

For EPN amplitude, a 2 (priming conditions) × 2 (horizontal electrodes: P/PO) × 4 (vertical electrodes: 5/6/7/8) × 2 (group) repeated measures ANOVA revealed a significant main effect of priming (*F*_(1,34)_ = 5.296, *p* = 0.028, $\eta_{\text{p}}^{2}$ = 0.135). That is, ER-related priming (*M* = 3.035, SD = 0.429) elicited larger EPN than ER-unrelated priming (*M* = 3.510, SD = 0.470). A significant main effect of vertical electrodes (*F*_(3,102)_ = 13.732, *p* < 0.001, $\eta_{\text{p}}^{2}$ = 0.288) was found, and the EPN at the right-side electrodes was larger than the left-side ones. The interaction between priming and group was marginally significant (*F*_(*1, 34*)_ = 3.862, *p* = 0.058, $\eta_{\text{p}}^{2}$ = 0.102). The main effects of horizontal electrodes (*F*_(1,34)_ = 0.202, *p* = 0.656) and group (*F*_(1,34)_ = 0.007, *p* = 0.935), however, were not significant.

To examine the interaction between priming and group, a further 2 (priming conditions) × 2 (horizontal electrodes: P/PO) × 4 (vertical electrodes: 5/6/7/8) repeated measures ANOVA was conducted within the HTA and the LTA group respectively. While the main effect of priming was not significant in the HTA group (*F*_(1,17)_ = 0.091, *p* = 0.767), it was significant in the LTA group (*F*_(1,17)_ = 6.617, *p* = 0.020, $\eta_{\text{p}}^{2}$ = 0.280; see Figure 3).

##### LPP Amplitude

**Figure 4:**
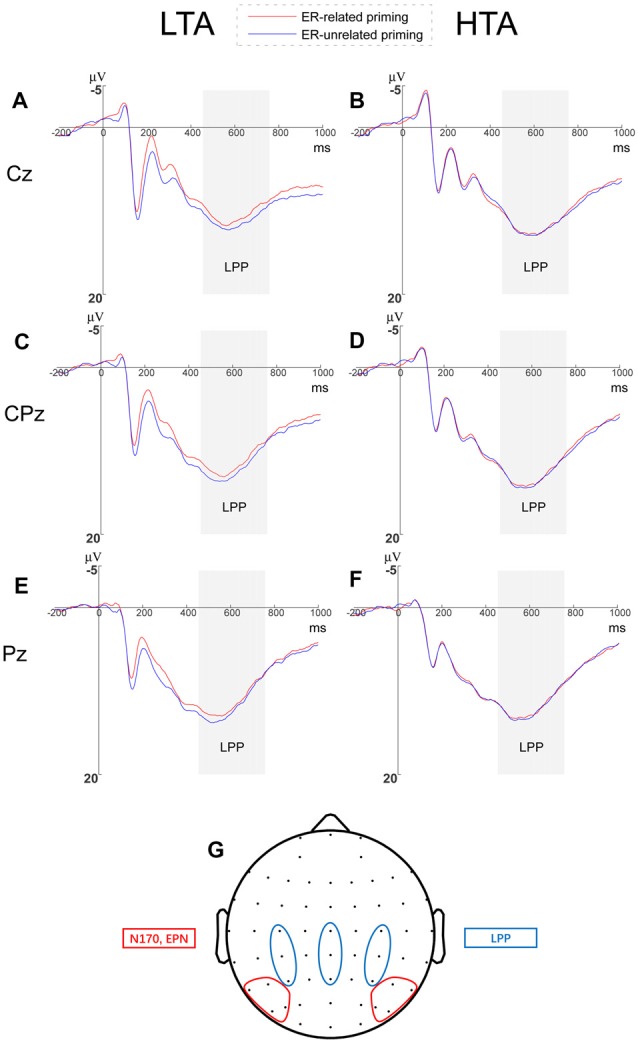
Grand averages of late positive potential (LPP) amplitudes and Topographical distribution. Grand averages of LPP elicited by threatening faces at Cz, CPz and Pz within **(A–C)** the LTA and **(D–F)** the HTA groups. **(G)** Topographical distributions diagram for all the analyzed event-related potential (ERP) components.

We analyzed LPP amplitudes by a 2 (priming conditions) × 3 (horizontal electrodes: C/CP/P) × 3 (vertical electrodes: 3/Z/4) × 2 (group) repeated measures ANOVA and found no significant main effects of horizontal electrodes (*F*_(2,68)_ = 11.553, *p* < 0.001, $\eta_{\text{p}}^{2}$ = 0.254) and vertical electrodes (*F*_(2,68)_ = 29.853, *p* < 0.001, $\eta_{\text{p}}^{2}$ = 0.468). We did observe that the LPP amplitude induced at central-parietal sites was larger than central and parietal sites (see Figure 4, a small topographical distribution diagram was presented in the Figure 4 as well). The main effects of priming (*F*_(1,34)_ = 0.013, *p* = 0.910) and group (*F*_(1,34)_ = 0.848, *p* = 0.364) were not significant.

### Correlations

**Figure 5:**
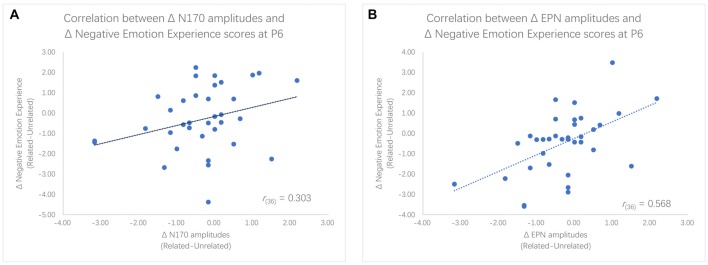
Scatter plots for the correlation between enlargement of N170 amplitudes and decreases in Negative emotion experience rating scores **(A)** as well as the correlation between enlargement of EPN amplitudes and decreases in rating scores **(B)**.

We found significantly different N170 and EPN amplitudes between the two priming conditions within the LTA group, but these differences were not significant within the HTA group. To further examine the relationship between ERP and behavior results, Pearson (*r*) correlation coefficients were computed between the negative emotion experience ratings and the N170 or EPN amplitudes. Given that the main effect of ordinal positions was not significant, we used the average of two ratings as the index of negative emotion experience. Results showed that decreases in negative emotion experience scores were significantly correlated with the enlargement of N170 amplitudes at P5 (*r*_(36)_ = 0.346, *p* = 0.039) and marginally significantly correlated with the enlargement of N170 amplitudes at P6 (*r*_(36)_ = 0.303, *p* = 0.072). Furthermore, decreases in negative emotion experience scores were significantly associated with greater EPN amplitudes at P6 (*r*_(36)_ = 0.568, *p* < 0.001), P8 (*r*_(36)_ = 0.436, *p* = 0.008) and PO8 (*r*_(36)_ = 0.615, *p* < 0.001; see Figure 5).

## Discussion

The current study investigated the potential deficits in implicit ER in HTA and LTA individuals. First, at the behavioral level we showed that implicit ER processing, in the form of a decrease in negative emotion experience scores, was induced by an ER-related priming condition during a subsequent task. This effect was especially prominent in the LTA group. Second, findings from the intention detection test confirmed that participants were not explicitly aware of the fact that ER priming was being used to modulate subsequent emotional experience. In other words, all ER processing involved in this study did indeed take place at the implicit level. Third, we found a non-significant main effect of the ordinal position of negative emotion experience scores, demonstrating that the priming effect was not decreased with time. As, observed modulations in ERP components could be considered as effective indexes of ER. Fourth, decreases in negative emotion experience scores were associated with enlargements of N170 and EPN amplitudes, thus indicating that these components could effectively index implicit ER. Finally, early N170 amplitudes, as well as the middle EPN, were enlarged by ER-related priming words in the LTA group but not in the HTA group. That is, a lack of enlargement of N170 and EPN amplitudes was observed in anxious individuals during the implicit ER processing, while the late stage processing (LPP) was not modulated by implicit ER goals in either group. Our results suggest that anxious individuals show deficits in implicit ER occurring over the early and middle stages of emotion processing.

The present study focused on implicit ER processing in anxiety. Three ERP components were chosen for indexing the time course of implicit ER: N170, EPN and LPP. We observed an enlarged N170 amplitudes within the LTA group but not the HTA group, as well as a significant correlation of its enlargement with decreases in negative emotion experience scores. This finding in the LTA group supports our first hypothesis and confirms the notion that an enlarged N170 could be considered as an index of implicit ER during the early stage of face processing. The absence of an enlarged N170 within the HTA group suggests a deficit of implicit ER in anxiety, as put forward in our second hypothesis. N170 is a well-known face-specific component (Bentin et al., 1996; Carmel and Bentin, 2002), however, there is some inconsistencies regarding its role in emotional processing. Some studies report no significant N170 differences between emotional expressions and neutral ones, suggesting that it reflects the encoding of facial configuration but not signals relating to emotion (Eimer et al., 2003; Ashley et al., 2004). Contrary to this view, some other studies have reported that N170 is sensitive to facial expression, whereby threatening or joyful faces elicited larger N170 amplitude than neutral ones (Batty and Taylor, 2003; Blau et al., 2007; Luo et al., 2010). Our finding regarding its role in implicit ER may provide some insight into the source of this discrepancy. For instance, varying levels of engagement in implicit ER may explain much of the heterogeneity found across these studies. Regarding the role N170 plays in the emotional face processing, it was described in a well-known face processing model that the facial structure encoding unit includes a view-center descriptions module and an expression-independent descriptions module, which might be isolated from each other (Bruce and Young, 1986). The view-center descriptions module provides information for the analysis of expression, and the expression-independent descriptions module provide information for the global configuration and of features. In the current study, facial expressions were homogeneous between both compared experimental conditions, and then the enhanced N170 could not reflect more elaborate emotion recognition processing. Consistent with both implicit ER (Mauss et al., 2007a) and face processing (Bruce and Young, 1986) models, we suggest that ER priming-induced enlargement of N170 observed in the present study represents attentional deployment. More specifically, ER goals facilitated facial structural encoding and deepened emotion-unrelated encoding (i.e., an implicit form of distraction). The absence of any enlargement of N170 amplitude within the HTA group might indicate a reduction in the ability to implicitly deploy attention (i.e., less efficient distraction) for individuals with anxiety.

In the present study, we were surprised to find a significant priming effect for the EPN amplitude within the LTA group, as well as a non-significant priming effect within the HTA group. Our hypothesis was formed on the basis of previous findings from a study using the PI paradigm (Wang and Li, 2017) and we expected the EPN to be unaffected during implicit ER processing within the LTA group. However, we also found that reductions in negative emotion experience scores were highly correlated with the enlargement of EPN amplitudes, which was not addressed in previous ER studies. The main difference of experimental task between in the current study and in the prior study is the amount of priming conditions. In the previous study (Wang and Li, 2017), healthy participants were primed with three conditions: control, Expression and Unrelated. Although the Expression priming condition was aimed at up-regulating the emotional response of participants, it failed to fulfill this intention and, consequently, only two priming conditions were included in the present study. We might speculate that the previously undiscovered priming effect of EPN might be due to the interference of the additional Expression priming condition on the statistical power.

Previous studies have observed a greater EPN induced by emotional stimuli than neutral stimuli during non-conscious processing (Schupp et al., 2004b; Pegna et al., 2008; Luo et al., 2010), regardless of whether the stimuli were scenes or faces (Junghöfer et al., 2001; Schupp et al., 2004a). Rellecke et al. (2012) found that angry expressions elicited a larger EPN than happy expressions, which was interpreted to show that the EPN amplitude reflects an automatic threat-privileged processing associated with affective stimuli evaluation. Conversely, other researchers have observed a more pronounced EPN for happy faces compared to angry ones (Bublatzky et al., 2014; Leleu et al., 2015). The existing results indicated that EPN could generally be regarded as a higher-level index of affective processing. However, only threatening expressions were included in the current study. These faces were homogeneous between both experimental conditions, so that the priming-induced enlargement of EPN amplitudes was more likely to reflect the cognitive representation instead of affective processing. Regarding its role in cognitive processing, previous study suggested that EPN reflects early selective attention in visual processing (Schupp et al., 2007), whereby the EPN amplitudes were observed by comparing emotional vs. neutral stimuli. In the present study, as shown by the statistically significant correlation, the EPN amplitudes were modulated by ER related priming words, suggesting another role EPN reflects in cognitive processing. Schupp et al. (2008) suggested that “EPN reflects a transitory processing period at which motivationally significant stimuli are ‘tagged’ for preferential processing in higher-order visual-associative brain areas.” Based on previous literature regarding neural projections, Leleu et al. (2015) put forward an explanation for this transitory “tag” period at the neural level. They proposed that EPN may index an initial integration of the specific emotional meaning that the visual processing shares emotional content through connections between multiple locations (i.e., the visual system, the amygdala, the orbitofrontal cortex, the insula and the somatosensory cortices). This process of tagging emotional stimuli should be regarded as the implicit appraisal process in Gross’s model of automatic emotional regulation (Mauss et al., 2007a; Mocaiber et al., 2010). Combined with our result, we can speculate that threatening stimuli could be “tagged” differently during implicit ER processing. Put another way, the observed enlargement of EPN amplitude may represent a change in appraisal at the implicit level. LTA individuals may have been able to efficiently “tag” the threatening expression as safe or non-dangerous when primed with ER-related words, thus leading to a decreased negative emotion experience. The lack of any EPN amplitude enlargement between the two priming conditions within the HTA group may indicate a deficit of implicit ER (a less effective change of implicit appraisal) in anxiety occurring during the middle stage of emotional processing.

In consistent with our hypothesis, the analysis of LPP amplitude revealed a non-significant main effect of priming with no anxiety-related impact. In addition, we found no correlation between LPP amplitudes and behavioral results. Our results seem contradictory to one from Mocaiber et al. (2010), which showed a reduced LPP within both HTA and LTA groups when individuals were given an “implicit reappraisal instruction” (i.e., “pictures do not correspond to real situations”). However, LPP has been considered by some researchers to represent the allocation of sustained resources of attention to emotional stimuli (Schupp et al., 2003, 2006; Yang et al., 2015; Yuan et al., 2015b), and hence, a frequently-used index in studies of explicit ER (Dennis, 2010). These results might involve conscious effort during ER processing, such that the fictitious condition could be considered an explicit “reappraisal” condition. Our result is consistent with the previous PI paradigm study in healthy adults (Wang and Li, 2017), which showed that LPP amplitudes were not influenced by any of the priming condition (e.g., control, expression). This suggests that implicit ER did not demand cognitive resources at the late stage of emotion processing, and that no anxiety-related deficit of implicit ER was found at this stage.

The present study provides neural evidence for a deficit of implicit ER in anxiety, however, our results should be interpreted in the light of several limitations. First, in the present study, we used subjective rating scores of negative emotion experience to reflect the emotional response of participants after regulation came into effect. However, given the fact that implicit ER is by definition a process during which participants were without conscious, intention, awareness, or deliberate control (Mauss et al., 2007a), explicit behavioral level indicators might not be effective, stable, or sensitive enough as outcome measurements. Some studies have proposed that some physiological measurements, such as heart rate, mean arterial blood pressure, cardiac output, or total peripheral resistance, would represent more objective indexes of emotion responses (Jackson et al., 2003; Mauss et al., 2007b). In the future, more physiological indexes should be adopted to detect the effect of ER. Second, the present study does suffer from a small sample size, it has led to some marginally significant findings (e.g., interaction between priming and group on the ERP findings). Another possible reason for the findings could be that our participants were recruited from local colleges with a high or low level of trait anxiety. It would be more insightful to conduct our research on patients. Third, in the present study, enlarged N170 and EPN were found to be associated with implicit ER processing. However, future work could provide further insight through the addition of a non-priming condition, while still implicit, because one may compare results perceived from this condition with existing studies (Rossignol et al., 2005; Frenkel and Bar-Haim, 2011; Walentowska and Wronka, 2012; Otoole et al., 2013; Morel et al., 2014; Chronaki et al., 2018). A non-priming condition could be adopted to attain a “baseline level” state of emotional face processing. Such a state would be comparable with related studies and would, in turn, provide more insight into implicit ER in anxiety.

## Conclusion

To sum up, in the present study we observed an implicit ER deficit among anxious individuals occurring in the early and middle stages of emotion processing (emotion perception, emotion recognition). This deficit was expressed as an absence of enlargements in N170 and EPN amplitudes. Furthermore, we suggest that both N170 and EPN are effective indexes of implicit ER: N170 reflects implicit attention deployment and EPN reflects change of implicit appraisal.

## Author Contributions

BL analyzed, interpreted the data and wrote the first draft of the manuscript. YW designed the study, collected and pretreated the data. XL generated the idea, designed the study, interpreted the data and wrote the first draft of the manuscript.

## Conflict of Interest Statement

The authors declare that the research was conducted in the absence of any commercial or financial relationships that could be construed as a potential conflict of interest.
